# Exploring the potential of eRNAs in cancer immunotherapy

**DOI:** 10.1016/j.omto.2022.10.009

**Published:** 2022-11-08

**Authors:** Lianxiang Luo, Xinming Chen

**Affiliations:** 1 The Marine Biomedical Research Institute, Guangdong Medical University, Zhanjiang, Guangdong 524023, China; 2 The Marine Biomedical Research Institute of Guangdong Zhanjiang, Zhanjiang, Guangdong 524023, China; 3 The First Clinical College, Guangdong Medical University, Zhanjiang, Guangdong 524023, China

## Main text

The recent success of RNA therapy for non-neoplastic diseases has fueled the development of various RNA-based cancer immunotherapies.^1^ Enhancer RNA (eRNA), short noncoding RNA (50–200 nucleotides) transcribed from the enhancer region, can affect anticancer drug resistance, which may be associated with one or more related tumor signaling pathways.^2^ This research has led to explorations of novel eRNA-based cancer treatments.

Bioinformatics analyses have revealed functional eRNA molecular subtypes in lung adenocarcinoma.^3^ However, the genome-wide eRNA landscape and its relevance to the immune microenvironment in hepatocellular carcinoma (HCC) remain undefined. In this issue, Bu et al. share a novel exploration of eRNA transcriptional regulation in HCC,^4^ which compelled us to examine how eRNAs regulate genes in cancer and the value of eRNAs in cancer immunotherapy.

### Transcriptional regulation of eRNAs

Human eRNAs are present in clusters associated with genes that are collaborating partners in protein complexes. This “functional cooperation” may be common during development to dictate transcription factor expression. Within clustered enhancers, redundancy and hierarchy can coexist in various combinations to guarantee functional cooperation on target genes.^5^

More research is needed to evaluate eRNA transcription as an independent criterion for predicting active enhancers and annotating non-transcribed enhancers. RNA surveillance pathways that block the decay of eRNAs can be exploited to detect less stable eRNAs, facilitating the characterization of putative non-transcriptional enhancers. Measuring eRNA levels can identify enhancer features in physiological and pathological conditions. Moreover, given their specificity for cell type and state, eRNAs may provide diagnostic and therapeutic targets.

Another important goal is better describing enhancer-promoter loops, preferably at the single-cell level. This will confirm the role of eRNAs in regulating circularization and further delineate the relationship between circularization, eRNAs, and gene transcription. Given the commonality between enhancers and promoters, we must determine their mutual regulation and underlying hierarchy in the loading of transcriptional machinery. A potential approach is systematically deleting promoters and enhancers to study their relationship in three-dimensional genomes.

### eRNA regulation of tumor-promoting genes

dCas9-based activators can induce eRNA production, which is positively correlated with mRNA expression downstream of the homologous promoter.^6^ For example, KHPS1 activates eRNA through triplex-dependent recruitment of epigenetic regulators and increases the expression of proto-oncogene SPHK1.^7^ Nuclear effector YAP1/TEAD4 interacts with ERα-binding enhancer, which shows enhanced binding after E2 stimulation to facilitate E2/ERα target gene induction and E2-induced oncogenic cell growth.^8^ eRNA SEELA facilitates histone recognition and oncogene transcription by enhancing interactions between chromatin and histone modifiers. The SEELA-SERINC2 axis also regulates cancer metabolism, potentially influencing leukemia progression.^9^

### eRNA regulation of MYC, P53, and BRD4

Many pivotal host genes of Kaposi’s sarcoma-associated herpesvirus (KSHV) latency, including proto-oncogene MYC, are controlled by super enhancers.^10^ In KSHV-infected primary effusion lymphoma (PEL), the eRNA-expressing MYC super enhancer is located downstream of MYC. Deletion or inhibition of eRNA expression significantly reduces MYC mRNA in PEL. Similarly, in colon cancer cells, the WNT/β-catenin-AHCTF1-CTCF-eRNA circuit enables the oncogenic super enhancer to promote cancer cell growth by coordinating the trafficking of the active MYC gene in the three-dimensional (3D) nuclear structure.^11^

p53 binding enhancer regions also have enhancer activity and interact with neighboring genes in chromosomes to transmit long-distance transcriptional regulation.^12^ Léveillé et al. explored p53-regulated enhancers by examining eRNA production.^13^ They found that long noncoding RNA (lncRNA) lncRNA activator of enhancer domains (LED) activates strong enhancers and that LED knockdown attenuates p53 function.

In human colorectal cancer cells, bromodomain-containing protein 4 (BRD4) is recruited to enhancers cooccupied by mutant p53 and supports the synthesis of enhancer-directed transcripts (eRNAs) in response to chronic immune signals. eRNA directly participates in gene regulation by regulating BRD4’s enhancer interaction and transcriptional function.^14^ BRD4 acts as an anti-breast cancer target to promote p63 and GRHL3 expression downstream of FOXO in breast epithelial cells.^15^ Interestingly, BRD4-dependent BIRC3 eRNA synthesis confers *Helicobacter pylori*-mediated apoptosis resistance.^16^

### Effects of eRNA on androgen receptor drive loops and glucocorticoid receptor

eRNA enhances gene activation via the androgen receptor (AR) drive loop. AR binds enhancer elements and regulates specific enhancer promoter loops, thus activating the AR regulatory network.^17^ Kallikrein-related peptidase 3 (KLK3) is an AR regulatory gene that encodes prostate-specific antigen (PSA). KLK3’s upstream enhancer generates a bidirectional enhancer in RNA, KLK3e. Functional antisense eRNAs negatively regulate the antisense ncRNAs of AR-related target gene sites in prostate cancer cells by recruiting DNMT1 on antisense enhancers in gene terminal regions and increasing methylation. Importantly, a chromatin double loop promotes the *cis* sense to the promoter and the antisense to the gene terminal region. Deleting antisense eRNA impairs adjacent mRNA expression and impedes cancer progression. Antisense eRNA expression correlates with biochemical recurrence and clinical marker PSA level in patient tissues.

Hormone therapy leads to enhancer landscape recombination in breast cancer cells.^18^ Upstream of oncogene DDIT4, glucocorticoid receptor (GR) binds to four sites that constitute hormone-dependent hyper enhancers. Three GR binding sites are required as hormone-dependent enhancers to differentially promote histone acetylation, transcription frequency, and burst size. By contrast, the fourth site inhibits transcription, but hormone treatment alleviates this inhibition. An estrogen-responsive RNA, P2RY2 eRNA (P2RY2e), participates in breast cancer development. CRISPR-Cas13a-mediated knockdown of P2RY2e inhibits the proliferation, invasion, and migration of bladder cancer cells; it may also weaken the tumor promoting effect of estrogen on bladder cancer.^19^

### eRNAs for cancer therapy

Uncontrolled enhancer activity with aberrant eRNA expression may promote tumorigenesis. Specific transcriptional eRNAs from active enhancers may serve as therapeutic targets in certain cancers.^20^ For instance, Bu et al. found that eRNAs in immune-related cluster 1 are enriched in immune infiltration and may respond to immune checkpoint inhibitors.^4^ However, to utilize eRNAs as therapeutic targets in cancer, we need to identify more eRNA classes and mechanisms. One technology, Cap Analysis of Gene Expression, can capture transcriptional eRNAs and identify enhancers with extremely high nucleotide resolution.^21^ Thus, clinical treatment prediction models constructed using eRNAs have broad prospects.

In general, eRNA plays a crucial role in regulating gene transcription. Additionally, eRNAs associated with hormone receptors play regulatory roles in multiple cancers. Mining the types and functions of eRNAs will contribute to the revolutionary application of eRNAs to cancer immunotherapy.
